# Coffee and caffeine intake and male infertility: a systematic review

**DOI:** 10.1186/s12937-017-0257-2

**Published:** 2017-06-24

**Authors:** Elena Ricci, Paola Viganò, Sonia Cipriani, Edgardo Somigliana, Francesca Chiaffarino, Alessandro Bulfoni, Fabio Parazzini

**Affiliations:** 1 Dipartimento della Donna, del Neonato e del Bambino, Fondazione IRCCS Ca’ Granda Ospedale Maggiore Policlinico, Via Commenda 12, 20122 Milan, Italy; 2 Reproductive Sciences Laboratory, Division of Genetics and Cell Biology, IRCCS Ospedale San Raffaele, Milan, Italy; 3 Department of Obstetrics, Gynecology and Neonatology, Fondazione IRCCS Ca’ Granda, Ospedale Maggiore Policlinico, Milan, Italy; 4 Unit of Obstetrics and Gynaecology, Humanitas San Pio X Hospital, Milan, Italy; 5 Department of Clinical and Community Science, University of Milano, Milan, Italy

**Keywords:** Systematic review, Male infertility, Semen quality, Sperm parameters, Fecundability, Coffee consumption, Caffeine, Life style, Risk factors

## Abstract

**Background:**

Semen quality, a predictor of male fertility, has been suggested declining worldwide. Among other life style factors, male coffee/caffeine consumption was hypothesized to influence semen parameters, but also sperm DNA integrity. To summarize available evidence, we performed a systematic review of observational studies on the relation between coffee/caffeine intake and parameters of male fertility including sperm ploidy, sperm DNA integrity, semen quality and time to pregnancy.

**Methods:**

A systematic literature search was performed up to November 2016 (MEDLINE and EMBASE). We included all observational papers that reported the relation between male coffee/caffeine intake and reproductive outcomes: 1. semen parameters, 2. sperm DNA characteristics, 3. fecundability. All pertinent reports were retrieved and the relative reference lists were systematically searched in order to identify any potential additional studies that could be included.

**Results:**

We retrieved 28 papers reporting observational information on coffee/caffeine intake and reproductive outcomes. Overall, they included 19,967 men. 1. Semen parameters did not seem affected by caffeine intake, at least caffeine from coffee, tea and cocoa drinks, in most studies. Conversely, other contributions suggested a negative effect of cola-containing beverages and caffeine-containing soft drinks on semen volume, count and concentration. 2. As regards sperm DNA defects, caffeine intake seemed associated with aneuploidy and DNA breaks, but not with other markers of DNA damage. 3. Finally, male coffee drinking was associated to prolonged time to pregnancy in some, but not all, studies.

**Conclusions:**

The literature suggests that caffeine intake, possibly through sperm DNA damage, may negatively affect male reproductive function. Evidence from epidemiological studies on semen parameters and fertility is however inconsistent and inconclusive. Well-designed studies with predefined criteria for semen analysis, subject selection, and life style habits definition, are essential to reach a consistent evidence on the effect of caffeine on semen parameters and male fertility.

## Introduction

Approximately 13% of the general reproductive age population is challenged with fertility problems, and male factors seem to contribute for up to 30% of them [1]. Semen quality, a predictor of male fertility, has been suggested declining worldwide [2–4]. Many factors have been proposed as causes of this decline, including life style habits and trends toward high-protein western–style diets. Thus, given the supposed impact of smoking [5], alcohol consumption [6], weight [7], physical activity [8, 9] and diet [10] on spermatogenesis, the relation between semen parameters and life style has become a topic of interest.

Caffeine (1,3,7-trimethylxanthine) is found in coffee, tea, soft drinks (particularly cola-containing beverages and energy drinks) and chocolate. It easily crosses biologic membranes, is rapidly distributed throughout the body and has been found in saliva, breast milk, the embryo and the neonate [11]. The caffeine molecule is easily absorbed by humans, having approximately 100% of bioavailability when taken by oral route and reaching a peak in the blood within 15–45 min after its consumption [12]. Caffeine has a number of biologic effects, including central nervous system stimulation, increased secretion of catecholamine, relaxation of smooth muscles and stimulation of heart rate. It is known to have both positive and negative effects on health. Whereas a moderate intake may confer a modest protective effect against some cardiovascular system diseases and on the metabolism of carbohydrates and lipids (including the various forms of arterial cardiovascular disease, arrhythmia, heart insufficiency, diabetes, liver disease [13] and even Parkinson’s disease [14]), excessive amounts may lead to deleterious health effects [12]. Of particular concern is the increasing consumption of energy drinks, that are rich in caffeine and very popular among young people [15].

Male coffee/caffeine consumption has been associated with high levels of testosterone and sex hormone binding globulin (SHBG) [16]. It has been hypothesized that caffeine alters Sertoli Cells glycolytic and oxidative profile, interfering with male’s reproductive potential [17]. However, the mechanism behind the possible harmful effect of caffeine is not well clarified. In both fetal and adult life, caffeine may act indirectly by impacting the hypothalamo-pituitary-gonadal-system or by a direct toxic effect on the germinative epithelium [17, 18]. Moreover, coffee consumption has been hypothesized to influence not only semen parameters, but also sperm DNA integrity. This aspect is of potential relevance, considering that human sperm DNA damage can also be determined by testicular or post-testicular injury including oxidative stress [19]. Available evidence indicates that semen samples containing a percentage of DNA fragmented cells above a critical threshold have a reduced level of pregnancy success [20, 21].

Therefore, to summarize the currently available information, we conducted a systematic review of epidemiological data from observational studies on the relation between coffee/caffeine intake and parameters of male fertility including semen quality, sperm ploidy, sperm DNA integrity, and time to pregnancy.

## Methods

The electronic databases MEDLINE (1966 to 2016) and EMBASE (1985 to 2016) were searched for “coffee” or “caffeine” or “cola” and “semen quality” or “sperm quality” or “semen parameters” or “sperm parameters” or “fecundability” or “male infertility” or “male fertility”. The search included English and Human as limits.

Data were extracted independently by two investigators. If multiple published reports from the same study were available, only the one with the most detailed information was included. Review articles were considered only if they also reported original data. We included all observational papers that reported the relation between male coffee/caffeine intake and reproductive outcomes: semen parameters, sperm DNA characteristics, fecundability. We did not exclude abstracts at congresses. All pertinent reports were retrieved and the relative reference lists were systematically reviewed in order to identify any other relevant studies.

### General limits of reviewed papers

Some methodological considerations should be underlined before presenting the results of this review. The identified studies are markedly different in quality of information, study design and categorization of caffeine exposure. We collected information on study design, characteristics of men enrolled in the studies, estimates of coffee/caffeine consumption and confounding factors accounted for in the analysis. These aspects should be considered in interpreting the results.

## Results

**Fig. 1:**
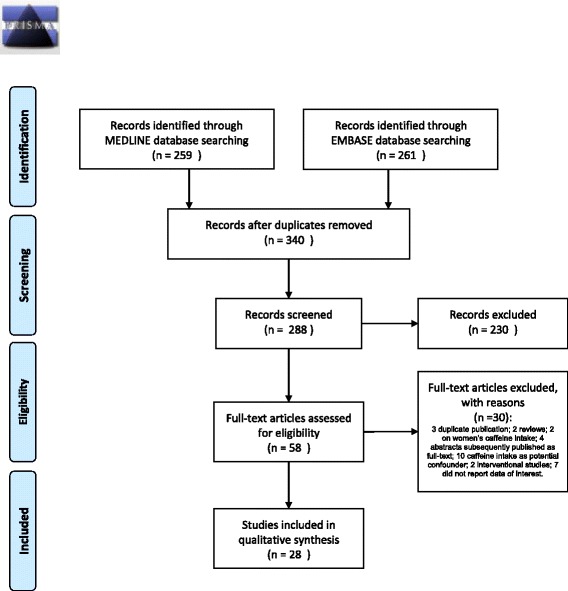
PRISMA flow diagram

Overall, we found 259 papers in MEDLINE and 261 in EMBASE (Fig. 1). The overlap was of 180 articles. Thus, 340 titles were reviewed. In this phase, 52 were excluded because reports of animal studies. Reviewing the abstracts, we excluded 98 laboratory studies, 39 reviews, 64 contributions just reporting data on women’s caffeine intake, 29 on other issues (contraception, urinary symptoms, editorials, correspondence about articles). Overall, 58 articles were candidates to be fully reviewed. Of them, 7 did not report data of interest; 4 were abstracts presented at congresses, subsequently published as full articles; 2 were reviews; 2 reported data only on women’s caffeine intake; 3 reported information about the same sample; 10 used caffeine intake as potential confounder but did not report the results; 2 were interventional studies. Twenty-eight articles were selected: 22 full-text [22–43] and 6 abstracts [44–49], that did not report detailed information on the method of exposure ascertainment. As such, they were included in the Tables but not commented in the Results. Overall, information on 19,967 men was reported. The main characteristics of the 28 ultimately selected papers are presented in Table 1.

**Table 1:** Main characteristics of studies on caffeine intake and male fertility

First author, year	Country	Number	Design	Setting	Outcome measure	Age (range or mean)
Full text						
Cole, 2006 [34]	Canada	41	Retrospective cohort	Obstetrics Department: planned pregnancies	Fecundability	22-45
Curtis, 1997 [27]	USA	2607	Retrospective cohort	Couples from farms in Ontario: planned pregnancies	Fecundability	17- > 30
Figà-Talamanca, 1996 [26]	Italy	72	Cross-sectional	Taxi drivers	Semen variables	43.2
Florack, 1994 [25]	The Netherlands	259	Prospective cohort	Non medical hospital workers’ partners	Fecundability	Not reported
Horak, 2003 [32]	Poland	179	Cross-sectional	Fertility clinic: healthy donors and men from infertile couples	Bulky DNA adducts in human sperm cells as a measure of DNA lesions	35.2
Jensen, 1998 [30]	Denmark	450	Prospective cohort	Trade union members	Fecundability	Not reported
Jensen, 2010 [38]	Denmark	2554	Cross-sectional	Young healthy men	Semen variables	18-22
Jurewicz, 2014 [40]	Poland	212	Cross-sectional	Healty men	Sperm aneuploidy	22-45
Klonoff-Cohen, 2002 [31]	USA	221	Prospective cohort	Fertility Clinic: infertile couples undergoing ART	Semen variables, clinical pregnancy, live birth	38.4
Kobeissi, 2007 [36]	USA	120/100	Case-control	Fertility Clinic: infertile couples	Cases suffered from impaired sperm count and function; controls were the fertile husbands of infertile women	38.6 cases/39.3 controls
Marshburn, 1989 [22]	USA	446	Cross-sectional	Infertile men	Semen variables	Not reported
Oldereid, 1992 [23]	Norway	252	Cross-sectional	Men attending a fertility laboratory	Semen variables	Not reported
Parazzini, 1993 [24]	Italy	97/105/120	Case-control	Fertility clinic	Cases of dyspermia; controls: 1. normospermic men of infertile couples; 2. Fertile men of unknown semen quality	Not reported
Radwan, 2016 [42]	Poland	286	Cross-sectional	Healthy men	DNA Fragmentation Index	22.7-44.8
Ramlau-Hansen, 2008 [37]	Denmark	344	Cross-sectional	Young men, sons of mothers in Healthy Habits for Two cohort	Semen variables	18-21
Robbins, 1997 [29]	USA	45	Cross-sectional	Young healthy men	Sperm aneuploidy	19-35
Schmid, 2007 [35]	USA	80	Cross-sectional	Non smoker healthy men	DNA damage	46.4
Sobreiro, 2005 [33]	Brazil	500	Cross-sectional	Candidates to vasectomy	Semen variables	35
Vine, 1997 [28]	USA	88	Cross-sectional	Healthy males	Sperm nuclear morphometric parameters	18-35
Wesselink, 2016 [43]	USA	2135	Prospective cohort	Couples planning a pregnancy	Fecundability	31.8
Wogatzky, 2012 [39]	Austria	1683	Cross-sectional	Fertility clinic: infertile couples	Semen variables	40.4
Yang, 2015 [41]	China	796	Cross-sectional	Young men	Semen variables	20 (median)
Published or only accessible as abstract						
Adelusi, 1998 [44]	Saudi Arabia	68/28	Case-control	Fertility Clinic: infertile couples	Sperm motility	Not reported
Al-Inany, 2001 [45]	Egypt	200	Cross-sectional	Fertility Clinic: infertile couples	Semen variables	23-45
Belloc, 2013 [46]	France	4474	Cross-sectional	Fertility Clinic: infertile couples	Semen variables, DNA fragmentation and chromatin decondensation	Not reported
Karmon, 2013 [47]	USA	166	Cross-sectional	Fertility Clinic: infertile couples	Semen variables	36.6
Karmon, 2014 [48]	USA	105	Prospective cohort	Fertility Clinic: infertile couples	Clinical pregnancy rate	37
Pecoraro, 2015 [49]	Italy	1134	Cross-sectional	Fertility clinic: infertile couples	Fertility	33.4 fertile/38.3 infertile

### Study design

Sperm variables were mainly evaluated within a cross-sectional design [22, 23, 26, 27, 32, 33, 37–39, 41, 45, 46]. The remaining studies were a case-control [44] and a prospective cohort [31]. One study evaluating sperm nuclear morphometric parameters [28] was cross-sectional.

All studies on DNA integrity [29, 35, 40, 42] had a cross-sectional design.

Rates of spontaneous fecundability were investigated by means of retrospective cohorts [27, 34] and prospective cohorts [25, 30, 43]. Success rates of assisted reproduction techniques (ART) were also studied using prospective cohort design [31, 47, 48]. Two case-control studies [24, 36] were also selected: men with dyspermia were compared to normospermic controls in the first study [24] and men with infertility were compared to men of known fertility in the second one [36].

### Data collection

Information on coffee and caffeine consumption was collected by different methods in various studies (Table 2). Frequently the caffeine intake was investigated as frequency of coffee consumption (cups/day). Some studies also collected information on tea and cola-containing beverage intake, as equivalent of 0.5 (tea) or 0.25 (cola) cups of coffee [28, 29, 35], while others recorded detailed information on various sources of caffeine and estimated the overall consumption [25, 27, 30, 31, 37, 38, 43]. Some authors were also able to analyze separately different sources of caffeine [27, 38, 41].

**Table 2:** Estimates of coffe/caffeine consumption, outcomes reported and confounding factors in the selected studies

First author, year	Estimates of caffeine (mg/serving)	Main findings	Confounding factors
Full text			
Cole, 2006 [34]	Not reported Caffeine drinks per month	Higher reported caffeine consumption was consistently, although not significantly, associated with longer time to pregnancy for both parents and the couple overall.	Intercourse frequency, mercury in blood
Curtis, 1997 [27]	coffee = 100 tea = 50 cola = 40 Level of daily intake	A slight decrease in fecundability among men was found when caffeine drinkers were compared with complete abstainers. Low (≤100 mg caffeine per day) versus high (>100 mg caffeine per day) consumption was also examined; no association with fecundability was observed using this cut-point. Consuming >3 cups of tea per day was associated with decreased fecundability.	Smoking, recent OC use, woman’s caffeine and age
Figà-Talamanca, 1996 [26]	Not estimated Cups of coffee per day	No consistent association between coffee consumption and sperm count, motility, morphology. High prevalence of atypical forms was observed among men drinking 1-3 cups of coffee/day, but not among those drinking >3.	Age, smoking and alcohol
Florack, 1994 [25]	coffee = 100 tea = 50 cola (375 ml) = 40 Level of daily intake	Heavy caffeine intake (>700 mg/day) among partners was negatively related to fecundability when compared with the lowest intake level (OR adjusted = 0.6, 95% CI, 0.3-0.97).	Smoking and alcohol, woman’s caffeine intake
Horak, 2003 [32]	Not estimated mL of coffee per day	No correlation between alcohol or coffee consumption and sperm DNA adducts	None
Jensen, 1998 [30]	coffee = 100 tea = 50 cola (100 ml) = 100 chocolate drink = 25 chocolate bar = 12.5 Level of daily intake	No adverse effect of caffeine among male smokers. Among nonsmokers, intake of more than 700 mg/d caffeine was associated with a Fecundability Ratio of 0.47 (95% CI 0.26–0.82) among males compared with nonsmokers whose daily caffeine intake was 0 to 299 mg/d. Among nonsmokers, we found no statistically significant associations between fecundability and intake of any specific source of caffeine, but a similar tendency was found for each source as for overall caffeine intake.	M and F: smoking, reproductive organs diseases, alcohol intake, age, BMI M: sperm concentration F: duration of menstrual cycle, use of OC as last method of birth control
Jensen, 2010 [38]	coffee = 117 tea = 70 cola (500 ml) = 70 chocolate drink = 5 chocolate bar = 7 Level of daily intake	Low (101–200 mg) to moderate (201–800 mg) daily caffeine consumption was not associated with a reduction in semen quality. Consumption of >800 mg of caffeine per day resulted in a nonsignificant reduction in semen quality. Semen volume, sperm concentration, total sperm count, and percentage of spermatozoa with normal morphology decreased among cola-drinking men compared with nondrinkers.	Fever >38C within the last 3 months, period of abstinence, BMI, in utero exposure to smoking, conditions found at the physical examinations, self-reported genital conditions, cryptorchidism
Jurewicz, 2014 [40]	Not estimated Days of coffee drinking/week	A positive relationship was found between coffee drinking everyday and the lack of chromosome X or Y, as well as coffee drinking 1–6 times per week and additional chromosome 18.	abstinence, age and past diseases
Klonoff-Cohen, 2002 [31]	coffee = 100 tea = 50 soda (can) = 100 chocolate drink = 4 chocolate bar = 7-18 (milk-dark) Level of daily intake	Male caffeine consumption had no relation with semen parameters, clinical pregnancy or achieving a livebirth. Analysed as a linear continuous predictor, was a significant risk factor for multiple gestation: OR = 2.2 (95% CI 1.1-4.4) and OR = 3.0 (95% CI 1.2-7.4) for men who increased their usual intake or intake during the week of initial visit by 100 mg/day.	Smoking, alcohol, years of schooling, partner’s age, race, indication to ART, number of attempt.
Kobeissi, 2007	Not estimated Cups of coffee per day	Cases had a slightly higher mean intake of coffee (cups/day 3.2 ± 4.7 vs 2.9 ± 4.7, p = 0.574). At the multivariate analisys, odds of caffeine intake for being infertile were 1.05 (95% CI 0.96-1.14 by 1 cup/day).	Family history of infertility, reproductive health index, smoking, soft drinks intake, occupational exposures, war exposure
Marshburn, 1989 [22]	Not estimated Cups of coffee per day	Coffee drinking was correlated with increases in sperm density and percentage of abnormal forms. Among non smokers, coffee drinkers had a higher percentage of motile sperm as compared to non-coffee drinkers.	Alcohol, smoking
Oldereid, 1992 [23]	Not estimated Cups of coffee per day	No relationship could be established between sperm concentration, motility and morphology, and the number of cups of coffee drank daily	None
Parazzini, 1993 [24]	Not estimated Cups of coffee per day	Adjusted rate ratios for dyspermia were significantly higher in men drinking 2-3 and ≥4 cups/day (reference 0-1), compared either to normospermic men (1.8 and 3.0 respectively) or men of unknown semen quality (RR 1.3 and 4.2 respectively).	Age, education, smoking, alcohol
Radwan, 2016 [42]	Not estimated Days of coffee drinking/week	Coffee drinking were not related with any of the examined parameters of sperm DNA damage and high DNA stainability	age, smoking, alcohol, past diseases, BMI, duration of couple’s infertility, abstinence, level of stress, cell phone use
Ramlau-Hansen, 2008 [37]	coffee = 100 tea = 50 cola (500 ml) = 50 Level of daily intake	Caffeine exposure did not seem to affect adversely the semen quality or the levels of inhibin B or FSH. No association between caffeine and sperm motility or morphology. Men with a high caffeine intake had about 14% higher concentration of testosterone than men with a low caffeine intake.	abstinence time, diseases of the reproductive organs, smoking, season, maternal smoking during pregnancy
Robbins, 1997 [29]	Equivalent of 8 oz. cup: Tea = 0.5*n Cola = 0.25*n Level of daily intake	No difference between groups (0, 1 or ≥2 cups/day) was observed in term of semen motility and morphology. Lower density was found in the light caffeine group. Caffeine was significantly associated with increased frequencies of sperm aneuploidy XX18 and XY18, diploidy XY18-18 and the duplication phenotype YY18-18	Age, smoking, alcohol
Schmid, 2007 [35]	Equivalent of 8 oz. cup: Tea = 0.5*n Cola = 0.25*n Level of daily intake	In tertiles of caffeine consumption, men with >308 mg of caffeine intake per day (equivalent to ∼ 2.9 cups of coffee) had ∼ 20% higher neutral % tail DNA than men with no caffeine intake (P = 0.01 unadjusted; P = 0.005 after adjusting for the covariates total kilocalorie intake and the history of urinary tract infections)	Vitamin C use, season, Kilocalories, urinary tract infections
Sobreiro, 2005 [33]	Not estimated Cups of coffee per day	Among patients not drinking coffee, progressive motility averaged 57.1%, whereas for the patients who consumed more than six cups of coffee per day, it averaged 62.4% (p for trend < 0.05). There were no significant differences in semen volume, sperm concentration or sperm morphology.	None
Vine, 1997 [28]	Equivalent of 8 oz. cup: Tea = 0.5*n Cola = 0.25*n Level of daily intake	No convincing evidence was found for associations between the means, standard deviations, or skewness of any of nine sperm nuclear morphometric parameters and caffeine exposure	Age, smoking, alcohol
Wesselink, 2016 [43]	Coffee = 135 Decaf.coffee = 5.4 Black tea = 40 Green tea = 20 White tea = 15 Soda = 23-69 Energy drinks = 48-280 Level of daily intake	Total caffeine intake among males was associated with fecundability (FR for ≥300 vs. <100 mg/day caffeine among males = 0.72, 95% CI = 0.54–0.96), although the association was not monotonic. With respect to individual beverages, caffeinated soda and energy drink intake were associated with reduced fecundability among males.	Age, ethnicity, education, smoking, alcohol, intercourse frequency, sleep duration, work time, partner’s caffeine intake
Wogatzky, 2012 [39]	Not estimated Cups of coffee per day	204 men out of 1321 drinking coffee had an intake of more than 3 cups of coffee per day. With respect to MSOME criteria, these patients revealed a marked tendency towards lower sperm quality.	None
Yang, 2015 [41]	Not estimated Cups of coffee per day	Coffee consumption was found to be associated with increased progressive and nonprogressive motility of 8.9% or 15.4% for subjects consuming 1–2 cups/wk or 3 cups/wk of coffee, respectively. Cola consumption appeared an association with decreased semen volume at 4.1% or 12.5% for 1–2 bottles/wk or 3 bottles/wk.	age, tobacco and alcohol consumption, duration of abstinence, BMI, coffee/cola/fried food/baked foods consumption
Published or only accessible as abstract			
Adelusi, 1998 [44]	Not reported	Frequent coffee drinking associated to higher sperm motility	n.d.
Al-Inany, 2001 [45]	Not reported	No association between coffee consumption and sperm parameters	n.d.
Belloc, 2013 [46]	Not reported	Among caffeine consumers, semen volume was slightly higher (3.2 ± 1.6 vs. 3.1 ± 1.6 ml, *p* < 0.01) as pH (*p* < 0.01), but concentration was lower (60.0 ± 90.7 vs. 69.6 ± 124.9 millions/ml, *p* < 0.01), azoospermia less frequent (2.7 vs. 4.4%, *p* < 0.01). No relationship was observed for motility and morphology, nor for DNA fragmentation and chromatin decondensation. In a multivariate model including age, results were confirmed for volume (*p* < 0.01), but not for concentration. Caffeine intake was associated with a lower risk of elevated fragmentation (OR = 0.92, 95% CI 0.92-0.99).	n.d.
Karmon, 2013 [47]	Not reported	Caffeine intake was not related to semen quality parameters	Alcohol, smoking
Karmon, 2014 [48]	Not reported	Male caffeine intake was negatively associated with clinical pregnancy per initiated cycle. Compared to men consuming <88 mg/day of caffeine, adjusted odds ratios (95% CI) for clinical pregnancy per initiated cycle were 1.4 (0.5-3.8), 1.7 (0.6-4.8), and 0.4 (0.1-1.0) for men consuming 88-168 mg/day, 169-264 mg/day, and ≥265 mg/day of caffeine, respectively.	Alcohol, smoking
Pecoraro, 2015 [49]	Not reported	Fewer fertile than infertile men were coffee drinkers (*p* = 0.003)	None

*BMI* body mass index, *OR* odds ratio, *RR* risk ratio, *CI* confidence interval, *FR* fecundability ratio, *MSOME* Motile Sperm Organelle Morphology Examination

## Caffeine and semen variables

Semen variables were considered in several papers, but not all of them specifically reported the relation with caffeine exposure [26, 31].

In a cross-sectional analysis, Figà-Talamanca et al. [26] studied a group of 201 taxi drivers, exploring the possible association between professional exposure and reproductive health. No consistent relation between coffee consumption and sperm count or motility was found. For sperm morphology, a high prevalence of atypical forms was found among men drinking 1-3 cups of coffee/day, but not among those drinking more than three.

In a prospective cohort study, Klonoff-Cohen et al. [31] collected information on timing and amount of caffeine intake by men and women undergoing in vitro fertilization, reporting the caffeine intake during their lifetime, 1 year prior the attempt, during the week of the initial clinical visit and during the week of IVF procedure. The association of male and female intake of beverages (coffee, tea, cola) and chocolates and multiple end points (including oocyte retrieval, sperm parameters, fertilization rate, multiple gestations, miscarriage rate, and live births) was evaluated. Accounting for potential confounders (Table 2), no relation was found between male caffeine intake and sperm count, motility or morphology.

## Volume

As shown in Table 3, no study found a significant relation between coffee/caffeine intake and semen volume. Although some studies suggested that men with the highest coffee consumption had lower semen volume as compared with those with less or no consumption [22, 37–39], this result was not statistically significant nor consistent throughout the studies. In the study by Yang et al. [41], it was even the opposite. In this regard, the only statistically significant result was found among cola consumers included in the study, as the higher the weekly cola intake, the lower was the volume [41]. A similar trend was observed by Jensen et al. [38], although their result was not statistically significant.

**Table 3:** Caffeine intake and sperm variables

Author	Number	Volume (mL)	Count (millions)	Concentration (millions/mL)	Motility (% motile forms)	Morphology (%)
Jensen, 2010 [38]		Median (IQR)	Median (IQR)	Median (IQR)	Median (IQR)	Median (IQR)^b^
Daily caffeine consumption (mg)^a^						
0-100	1164	3.2 (2.3-4.3)	146 (65-257)	46 (22-80)	66 (57-74)	6.5 (3.3-8.5)
101-200	521	3.2 (2.4-4.1)	133 (62-242)	42 (20-78)	67 (58-74)	7.0 (4.3-9.5)
201-800	657	3.2 (2.4-4.1)	149 (70-260)	47 (23-84)	68 (57-74)	6.5 (3.5-9.5)
>800	63	3.0 (2.1-4.1)	133 (68-192)	41 (26-64)	66 (57-74)	5.5 (3.3-9.3)
Marshburn, 1989 [22]		Mean (SE)		Mean (SE)	Mean (SE)	Mean (SE)^c^
Coffee cups per day						
0	166	3.0 (0.1)		76.7 (3.7)	59.0 (1.5)	28.0 (0.8)
1-3	198	3.1 (0.1)		89.1 (3.8)	62.0 (1.2)	28.0 (0.7)
≥4	82	2.7 (0.8)		**81.4 (5.8)**	57.0 (2.5)	**31.0 (1.4)**
Oldereid, 1992 [23]				Mean (SE)	Mean (SE)^d^	Mean (SE) ^c^
Coffee cups per day						
0	45			69.5 (9.6)	20.1 (2.1)	58.5 (3.0)
1-5	133			87.8 (7.1)	22.7 (1.4)	54.2 (1.8)
≥6	60			82.1 (9.9)	22.1 (2.1)	56.8 (2.7)
Ramlau-Hansen, 2008 [37]		Median (IQR)	Median (IQR)	Median (IQR)	Median (IQR)	Median (IQR) ^b^
Daily caffeine consumption (mg)^a^						
0-25	139	2.8 (2.3-3.8)	118 (50-206)	34 (18-78)	69 (60-76)	5.5 (3.0-8.5)
50-125	143	3.3 (2.1-4.1)	113 (39.288)	44 (22-90)	69 (63-77)	5.0 (3.0-8.0)
175-1075	62	2.5 (2.2-3.7)	145 (74-351)	44 (21-96)	71 (60-77)	6.8 (4.0-10.0)
Sobreiro, 2005 [33]		Mean (SE)		Mean (SE)	Mean (SE)	Mean (SE) ^b^
Coffee cups per day						
0	Nd	2.7 (1.5)		110.8 (79.7)	**57.1 (16.2)**	17.3 (8.2)
1-3	Nd	2.6 (1.4)		113.6 (82.0)	**60.7 (14.6)**	17.5 (10.0)
4-6	Nd	2.7 (1.3)		111.0 (94.8)	**61.2 (15.5)**	17.9 (8.3)
≥6	Nd	2.7 (1.7)		127.2 (82.3)	**62.4 (16.0)**	18.0 (9.2)
Wogatzky, 2012 [39]		Mean (SD)	Mean (SD)	Mean (SD)	Mean (SD)^e^	
Coffee cups per day						
<3	1479	2.7 (1.5)	58.0 (91.2)	23.1 (28.9)	4.9 (7.9)	
≥3	204	2.6 (1.5)	63.5 (66.9)	25.8 (31.5)	4.3 (8.1)	
Yang, 2015 [41]		Median (5^th^ and 95^th^ percentile)	Median (5^th^ and 95^th^ percentile)	Median (5^th^ and 95^th^ percentile)	Median (5^th^ and 95^th^ percentile)	Median (5^th^ and 95^th^ percentile)^b^
Coffee cups per day						
0	605	3.4 (1.6-6.8)	187 (37-626)	54 (13-200)	**55 (29-81)**	8.3 (4.0-13.9)
1-2	154	3.1 (1.4-5.9)	170 (39-628)	55 (14-183)	**59 (28-85)**	8.7 (4.5-14.8)
≥3	35	3.6 (1.5-7.4)	190 (49.781)	52 (21-226)	**60 (27-92)**	7.7 (3.9-13.0)
Cola						
Jensen, 2010 [38] Weekly cola consumption (0.5 L bottles)		Median (IQR)	Median (IQR)	Median (IQR)	Median (IQR)	Median (IQR)^b^
0	379	3.3 (2.4-4.5)	**171 (75-295)**	**50 (25-89)**	66 (57-73)	**8.0 (5.0-10.5)**
1-7	1759	3.2 (2.3-4.2)	**143 (65-254)**	**45 (22-80)**	67 (55-74)	**6.0 (3.5-9.5)**
-14	262	3.1 (2.4-4.1)	**138 (71-241)**	**47 (23-76)**	69 (58-76)	**6.0 (3.5-9.0)**
>14	93	3.0 (2.2-4.0)	**102 (42-197)**	**35 (17-66)**	66 (58-73)	**7.0 (5.0-10.0)**
Yang, 2015 [41] Weekly cola consumption (0.55 L bottles)		Median (5^th^ and 95^th^ percentile)	Median (5^th^ and 95^th^ percentile)	Median (5^th^ and 95^th^ percentile)	Median (5^th^ and 95^th^ percentile)	Median (5^th^ and 95^th^ percentile)^b^
0	273	**3.6 (1.7-6.6)**	**209 (40-761)**	57 (15-211)	**54 (28-80)**	8.5 (4.4-13.5)
<3	404	**3.4 (1.5-6.9)**	**175 (37-593)**	52 (14-184)	**57 (30-84)**	8.4 (3.9-15.0)
≥3	117	**3.1 (1.4-5.9)**	**154 (35-505)**	56 (11-158)	**71 (28-91)**	7.9 (4.3-13.9)

*IQR* interquartile range, *SE* standard error, *SD* standard deviation

Bold results are statistically significant

a: coffe, tea, chocolates

b: morphologically normal forms

c: abnormal forms

d: progressive motile

e: grade A motility

## Count

No relation was observed between coffee/caffeine consumption and total sperm count. The lack of effect was probably true, as no dose-response gradient was present. On the contrary, in two studies [38, 41] cola intake was found consistently associated with lower sperm count.

## Concentration

Results regarding sperm concentration were similar to those on total count; no significant difference was found in relation to coffee intake. The only exception was represented by the study of Marshburn et al. [22], that observed that in men with the highest intake (4 or more cups of coffee per day), the concentration was higher as compared to men who did not drink coffee at all, but was lower as compared to men drinking 1-3 cups of coffee per day.

Consistently with the findings reported for volume and total count, Jensen et al. [38] reported a significantly lower concentration in men with higher cola consumption. On the contrary, men observed by Yang et al. [41] had both lower volume and counts as cola intake increased, but no consistent trend emerged as regard to concentration.

## Motility

Motility was reported as total or progressive motility (Table 3). Most studies did not report any significant difference throughout the categories of coffee/caffeine consumption, whereas two [33, 41] observed an increasing percentage of motile sperm in men with the highest intake.

The same result was found by Yang et al. [41] in men drinking cola beverages, but this finding did not emerge in the study from Jensen et al. [38].

## Morphology

Morphology was reported as percentage of normal [33, 37, 38, 41] or abnormal [22, 23] forms. Only Marshburn et al. [22] reported a significantly higher proportion of abnormal forms in men drinking 4 or more cups of coffee per day (31% versus 28% in other categories of intake). In line, a lower percentage of normal forms was found in high-quantity cola drinkers [38, 41]. This difference resulted statistically significant in the study by Jensen et al. [38].

Vine et al. [28] found no consistent evidence for associations between the means, standard deviations, or skewnesses of any of sperm nuclear morphometric parameters (size, shape, stain and texture) and caffeine intake.

## Caffeine and DNA damage

Jurewicz et al. [40] and Robbins et al. [29] focused specifically on the relation between life style and sperm aneuploidy.

In particular, Robbins et al. [29] investigated caffeine effects on sperm aneuploidy within the context of potential confounding or interaction effects of alcohol and smoking. Caffeine intake, measured as coffee cup equivalents per day, demonstrated a significant linear association with increasing chromosomal abnormalities (XX18, XY18, YY18-18, XY18-18), after adjusting for the continuous variables alcohol, age and urine cotinine (as a marker of smoking).

Jurewicz et al. [40] also studied aneuploidy and diploidy in a group of healthy men. The average frequencies of aneuploidy for the specific chromosomes studied in this group were: XX 0.02%, YY 0.01%, XY 0.10% and 18-18 0.04%. The authors found a positive relationship between everyday coffee drinking and the lack of chromosome X or Y (p = 0.013), as well as coffee drinking 1–6 times per week and additional chromosome 18 (p = 0.026). This association persisted after accounting for factors known or suspected to affect aneuploidy (age, alcohol intake and cigarette smoking).

Besides aneuploidy, DNA integrity represents an extremely important parameter indicative of male infertility and of potential outcome of ART. A recent paper by Radwan et al. [42] failed to find evidence of a relation between DNA fragmentation evaluated by sperm chromatin structure assay and coffee drinking, in 286 healthy men. Coffee drinking was not related to any of the examined parameters of sperm DNA damage and DNA stainability, including the percentages of DNA fragmentation index (DFI), the medium DNA fragmentation index (M DFI), the high DNA fragmentation index (H DFI) and the high DNA stainability index (HDS—percentage of immature sperms).

Schmid et al. [35] also investigated the association between coffee and DNA damage in 80 healthy non-smokers, by sperm Comet analyses, and concluded that, independently of age (older men have increased sperm DNA damage), men with substantial daily caffeine consumption have increased sperm DNA damage associated with double-strand DNA breaks.

In line, Horak et al. [32] investigated the accumulation of DNA adducts as a biomarker of exposure to chemical mutagens. A DNA adduct represents a segment of DNA bound to a cancer-causing chemical substance. DNA repair mechanisms induced by several chemicals and radiation occur early during spermatogenesis, but not in mature spermatids and spermatozoa [50], raising the possibility of accumulation of non-repaired DNA damage during spermiogenesis. The association among DNA adducts, fertility and life style habits has been addressed, and a significant negative correlation between presence of DNA adducts and sperm concentration or motility was found among patients with an impaired fertility. However, no correlation between coffee consumption and sperm DNA adducts could be detected.

In this regard, it should be considered that controversies among studies might be related to the various methods of DNA damage evaluation. Sperm DNA fragmentation tests generally show moderate correlation to each other [51]. None of them provide specific information on the nature and severity of the DNA damage, and it is still unclear which of these tests is preferable to optimize clinical decision-making.

## Caffeine and risk of dyspermia

Two case-control studies [24, 36] compared coffee intake between men with impaired fertility and fertile controls. Parazzini et al. [24] investigated cases of men with unexplained dyspermia, comparing them with two control groups, from the same clinic where cases were selected: men of infertile couples with negative work-up for any disease affecting fertility, and fertile men of unknown semen quality, that were partners of women who gave birth (at term) to healthy infant. In this study, dyspermia was defined as low concentration (between 5 and 10*10^6^ sperm/mL), progressive motility <30%, <30% typical forms, leukocytes <1*10^6^/mL, and no sperm agglutination. Dyspermia risk increased with the number of coffee cups drank per day (reference category 0-1 cup): the relation was significant if cases were compared to fertile men (multivariate odds ratio (OR) 1.3 for 2-3, 4.2 for ≥4 cups, chi-square for trend *p* < 0.001), as well as versus normospermic men of infertile couples (multivariate OR 1.8 for 2-3, 3.0 for ≥4 cups, chi-square for trend p = 0.005).

In the study published by Kobeissi et al. [36], cases suffered from oligozoospermia, asthenozoospermia, teratozoospermia or azoospermia; controls were the fertile husbands of infertile women. Mean daily coffee consumption was slightly, but not significantly, higher in cases than in controls, and in the multivariate analysis, including several potential confounding variables, the OR for coffee intake per day (by 1 additional cup of coffee) was 1.05 (95% confidence interval (CI) 0.96-1.14).

## Caffeine and time to planned pregnancy

In five cohort studies the endpoint was time to planned pregnancy [25, 27, 30, 34, 43].

In a prospective cohort including women working in nonmedical function at 39 Dutch hospitals and their partners, Florack et al. [25] analyzed the relation between caffeine intake (from coffee, tea and cola) and fecundability. Men with low or moderate caffeine intake did not differ, but those with a high level were more likely to experience a reduction in fecundability. Including all the relevant potential confounders, men who drank 4-7 caffeine drinks per day had an OR of 0.8 (95% CI 0.5-1.5) and those who drank 8 or more an OR of 0.6 (95% CI 0.3-0.97), as compared to <3 caffeine drinks per day.

Curtis et al. [27] analyzed data from the Ontario Farm Family Health Study (retrospective cohort), to evaluate if smoking, alcohol and caffeine intake affected fecundability ratio (FR), defined as the fecundability for the exposed group divided by that of the unexposed group. Only planned pregnancies were selected for this analysis, thus considering 2607 pregnancies among 1277 couples. Sources of caffeine were considered coffee, tea and cola containing beverages. Among men, a slight decrease in fecundability was found when caffeine drinkers were compared with complete abstainers; however, because 96% of the respondents reported some caffeine consumption, low (<100 mg caffeine per day) versus high (>100 mg caffeine per day) consumption was also examined; no association with fecundability was observed using this cut-point. There was no dose-response gradient for caffeine consumption, nor were there interactions between women's and men's caffeine consumption or between caffeine consumption and cigarette smoking. Thus, the study failed to find any relation between overall caffeine consumption and fecundability. Consumption of each of the three beverages was also analyzed. For male coffee, tea and cola drinkers, there was no overall association with fecundability. However, consuming more than three cups of tea per day was associated with decreased fecundability (FR = 0.85, 95% CI 0.69-1.05), suggesting that the effect, if true, was due to constituents other than caffeine.

In Denmark, Jensen et al. [30] recruited 430 couples without previous reproductive experience, who intended to discontinue contraception in order to become pregnant. Couples were enrolled among union trade members, who were 20–35 years old, lived with a partner, and had no children. Analyzing intake in strata of smoking habits, the authors found no adverse effect of caffeine among male smokers. However, among nonsmokers, caffeine intake > 700 mg per day was associated with a FR = 0.47 (95% CI 0.26–0.82) among males, compared with nonsmokers with 0-299 mg caffeine daily intake. No statistically significant association between fecundability and intake of any specific source of caffeine was observed, but a similar tendency was found for each source as well as for overall caffeine intake.

The objective of Cole et al. [34] was to retrospectively investigate the effects of maternal and paternal measures of persistent toxic substances on time to pregnancy among 41 couples from a general population, where the woman was at first trimester of pregnancy, taking into account other known factors affecting fecundability (the probability per month of becoming pregnant). The crude fecundability OR for paternal caffeine consumption was 0.49 (95% CI 0.20-1.20) for intake >52 drink/month (median intake) and for couple consumption was 0.73 (95% CI 0.30-1.74). In the multivariate model including all significant variables, couple, but not male, caffeine consumption above median, as compared to below median, remained significantly associated (OR 0.25, 95% CI 0.10-0.63) to prolonged time to pregnancy.

Wesselink et al. [43] studied the association between female and male preconception caffeine intake and fecundability in a North American prospective cohort study of 2135 pregnancy planners. In this study, male caffeinated soda intake showed an inverse dose-response relation with fecundability (1 and ≥2 vs. 0 cans/day: FR 0.77, 95% CI 0.56-1.05 and FR 0.72, 95% CI = 0.46–1.11, respectively). Male energy drink intake was also associated with reduced fecundability (≥1 vs. 0 cans/day: FR 0.46, 95% CI 0.21-0.98), whereas caffeinated coffee, black tea, and green tea were not. Decaffeinated coffee (>0 vs. 0 cups/day: FR 0.73, 95% CI 0.46-1.17) and herbal/decaffeinated tea (≥1 vs.0 cups/day: FR 0.64, 95% CI 0.32-1.31) were associated with slightly decreased fecundability, whereas decaffeinated soda was not (>0 vs. 0 cans/day: FR 0.90, 95% CI 0.70-1.16).

## Caffeine and ART

A prospective cohort study by Klonoff-Cohen et al. [31] investigated the rate of successful ART among coffee drinkers. Even if detailed results were not reported, the authors stated that male caffeine consumption had no effect on fertilization, pregnancy or live birth delivery. On the contrary, when caffeine was analyzed as a continuous variable, it represented a risk factor for multiple gestations. An increase of male caffeine intake by an additional 100 mg/day significantly increased the risk of multiple gestations by 2.2 times (95% CI 1.1-4.4) for usual consumption during lifetime; and by 3.0 times (95% CI 1.2-7.4) for intake during the week of initial clinic visit.

## Discussion

This systematic review focused on the relation between coffee/caffeine intake and male infertility using three main outcomes: semen variables, sperm DNA damage and time to pregnancy. In most studies, semen parameters did not seem affected by caffeine intake, at least caffeine from coffee, tea and cocoa drinks. Conversely, some studies suggested a negative effect of cola-containing beverages and caffeine-containing soft drinks on volume, count and concentration. As regards sperm DNA defects, caffeine intake seemed associated with aneuploidy and DNA breaks, but not with other markers of DNA damage. Finally, coffee drinking was associated to prolonged time to pregnancy in some, but not all, studies.

The extreme heterogeneity in exposure measurement, study design, and studied outcomes currently hampers the possibility to draw a definite figure on the relation between coffee/caffeine intake and male infertility. Meta-analyses, in particular, cannot be drawn.

Caffeine exposure has been assessed asking the usual daily or weekly intake of coffee alone [22–24, 26, 32–34, 36, 39–42], or of different sources of caffeine, such as tea, cola beverages [25, 27–29, 35, 37]; cocoa drink and chocolate bar intakes were also collected [30, 31, 38], as well as white tea, black tea, and decaffeinated coffee in a study [43]. It has been suggested that using coffee intake as a surrogate measure for caffeine exposure may severely underestimate its intake. Although coffee is the main source of caffeine, assessment of coffee alone is likely to underrate caffeine intake and, subsequently, its role as a risk factor. On the other hand, measurement of coffee, tea, and cola soft drink seemed to sufficiently approximate caffeine intake [52].

Studies on time to pregnancy had a cohort design, retrospective in two [27, 34] and prospective in three cases [25, 30, 43]. Both approaches are prone to bias: retrospective cohorts have a major limitation in the timing of assessment of caffeine intake, relative to the time of trying to become pregnant and thus subject to recall bias. However, since caffeine consumption is a behavior that often fluctuates over time, the information collected at enrolment in a prospective cohort might be outdated at the time of outcome assessment.

Case-control studies are also subjected to recall bias, as well as to selection bias. Careful choice of controls and information on confounding factors are usually included in good quality studies as those here presented [24, 36], but underlying confounders not accounted for may exist. Studies on the relation between coffee/caffeine and semen parameters [22, 23, 26, 28, 29, 33, 37–39, 41] or DNA damage [29, 32, 35, 40, 42] had a cross-sectional design, that shares the same risks of biases of case-control studies.

Then, results may be conditioned by some confounders that were not systematically and properly taken into account. This limitation may explain the significant associations observed in some but not all studies. Noteworthy is the recent emerging role of stress and diet in male infertility [53–55]. Coffee and caffeinated beverage consumption may actually be associated with peculiar diet patterns or life style habits, and it is difficult to disentangle spurious from potentially causal associations. For instance, the association found between semen variables and caffeine-containing soft drinks, but not with caffeine intake, may suggest a confounding effect. In other words, soft drinks rather than caffeine may be detrimental. The associations found for high quantity cola drinkers could not be attributed to the caffeine content in cola, which was not high; a less healthy life style among these men may explain the finding. In fact, some caffeinated beverages could affect fertility through mechanisms that do not involve caffeine. Cola-containing beverage intake, for instance, could cause subfertility through increased risk of insulin resistance, metabolic syndrome and weight gain [56–58]. Accordingly, male soda intake in general (and thus not only cola) has been shown to deleteriously affect sperm characteristics [59].

As regards study quality, in most published articles the relation between caffeine intake and reproductive outcomes was accounted for potential or well established confounding factors, as intercourse frequency, smoking, reproductive organs diseases, alcohol intake, age and BMI. However, the effect of residual unmeasured confounders cannot be excluded.

Finally, with regard to the analysis of time to pregnancy or successful ART rate, a neglected but potentially crucial aspect is the potential confounding effect of women’s fertility. Data from studies on spontaneous or ART-mediated infertility are actually exposed to the risk of women-related confounders, because the partners of a couple share at least in part the life style. If a pattern is detrimental to women but not to men fertility, one may found a spurious association in men.

In this complex methodological scenario, biological plausibility plays a critical role to speculate on potential causal relation, requiring some discussion.

Aneuploidy is an abnormality in the chromosome number and is the most prevalent type of genetic abnormality and a major type of chromosome aberration in humans. Meiosis that occurs continuously in men is a crucial and delicate event. In fact, different environmental and life style factors may interfere with the normal disjunction of sister chromatids/chromosomes during meiosis, thus resulting in aneuploidy. As many as 20% of human conceptions are believed aneuploid, but only 1/300 births, suggesting that aneuploidy plays a major role in pregnancy loss. A substantial proportion of aneuploidy occurring in embryos and newborns is of paternal origin [60]. In this regard, the evidence emerging from our review, that mainly supports an association between caffeine intake and sperm aneuploidy and DNA damage, deserves particular consideration. Noteworthy, the inconsistencies among available evidence are not surprising and may be explained by methodological issues. In fact, a burning debate currently exists on the use of sperm DNA damage tests in the infertility work-up of men, because of poor reproducibility [19, 20]. The availability of numerous assays to measure sperm DNA damage and the lack of well-designed comparative diagnostic studies have hampered, up to now, standardization and the full understanding of the clinical implications of sperm DNA damage [20].

On the other hand, most published results failed to find an association between semen variables and caffeine intake, at least caffeine from coffee, tea and coca drinks. Spermatogenesis is a complex process that is also very sensible to external agents. Semen quality constitutes a health benchmark and an important instrument for epidemiological studies of environmental impact [61]. However, well-defined criteria of what constitutes a development suitable model for the research process in studies of semen quality have been only recently developed [62]. Therefore, as there have been no specific standards for the appraisal of studies concerning semen quality until recently, biased results deriving from the older studies, in which quality controls were completely lacking, may have lead to erroneous conclusions potentially contributing to the heterogeneity observed. A stringent use of semen analysis criteria has been strongly advocated for future studies in order to overcome inconsistencies [63].

## Conclusions

The published evidence suggests that caffeine intake, possibly though sperm DNA damage, may negatively affect male reproductive function. Evidence from epidemiological studies on semen parameters and male fertility is however inconsistent and inconclusive. Well-designed studies, with predefined criteria for semen analyses and for subject selection, as well as defining life style habits, are essential to reach a strong evidence on the effect of caffeine on semen parameters and male fertility.

## Abbreviation

ART: Assisted reproduction techniques
BMI: Body mass index
DNA: Deoxyribonucleic acid
FR: Fecundability ratio
IQR: Interquartile range
MSOME: Motile Sperm Organelle Morphology Examination
OR: Odds ratio
RR: Risk ratio
SBHG: Sex hormone binding globulin
SD: Standard deviation
SE: Standard error

## References

[1] Nyboe Andersen A, Carlsen E, Loft A (2008). Trends in the use of intracytoplasmatic sperm injection marked variability between countries. Hum Reprod Update.

[2] Carlsen E, Giwercman A, Keiding N, Skakkebaek NE (1992). Evidence for decreasing quality of semen during past 50 years. BMJ.

[3] Swan SH, Elkin EP (1999). Declining semen quality: can the past inform the present?. Bioessays.

[4] Skakkebaek NE, Rajpert-De Meyts E, Buck Louis GM, Toppari J, Andersson AM, Eisenberg ML, et al. Male Reproductive Disorders and Fertility Trends: Influences of Environment and Genetic Susceptibility. Physiol Rev. 2016;96:55–97. doi:10.1152/physrev.00017.2015. 10.1152/physrev.00017.2015 PMC4698396 26582516

[5] Lotti F, Corona G, Vitale P, Maseroli E, Rossi M, Fino MG, et al. Current smoking is associated with lower seminal vesicles and ejaculate volume, despite higher testosterone levels, in male subjects of infertile couples. Hum Reprod. 2015;30:590–602. doi:10.1093/humrep/deu347. 10.1093/humrep/deu347 25567620

[6] Ricci E, Al Beitawi S, Cipriani S, Candiani M, Chiaffarino F, Viganò P (2017). Semen quality and alcohol intake: a systematic review and meta-analysis. RBMO.

[7] Tsao CW, Liu CY, Chou YC, Cha TL, Chen SC, Hsu CY (2015). Exploration of the association between obesity and semen quality in a 7630 male population. PLoS ONE.

[8] Gaskins AJ, Afeiche MC, Hauser R, Williams PL, Gillman MW, Tanrikut C (2014). Paternal physical and sedentary activities in relation to semen quality and reproductive outcomes among couples from a fertility center. Hum Reprod.

[9] Gaskins AJ, Mendiola J, Afeiche M, Jørgensen N, Swan SH, Chavarro JE (2015). Physical activity and television watching in relation to semen quality in young men. Br J Sports Med.

[10] Buhling KJ, Laakmann E (2014). The effect of micronutrient supplements on male fertility. Curr Opin Obstet Gynecol.

[11] Monteiro JP, Alves MG, Oliveira PF, Silva BM (2016). Structure-Bioactivity Relationships of Methylxanthines: Trying to Make Sense of All the Promises and the Drawbacks. Molecules.

[12] Sepkowitz KA (2013). Energy drinks and caffeine-related adverse effects. JAMA.

[13] Cano-Marquina A, Tarín JJ, Cano A (2013). The impact of coffee on health. Maturitas.

[14] Qi H, Li S (2014). Dose-response meta-analysis on coffee, tea and caffeine consumption with risk of Parkinson's disease. Geriatrics Gerontology Int.

[15] Reissig CJ, Strain EC, Griffiths RR (2009). Caffeinated energy drinks—a growing problem. Drug Alcohol Depend.

[16] Svartberg J, Midtby M, Bønaa KH, Sundsfjord J, Joakimsen RM, Jorde R (2003). The associations of age, lifestyle factors and chronic disease with testosterone in men: the Tromsø Study. Eur J Endocrinol.

[17] Dias TR, Alves MG, Bernardino RL, Martins AD, Moreira AC, Silva J (2015). Dose-dependent effects of caffeine in human Sertoli cells metabolism and oxidative profile: relevance for male fertility. Toxicology.

[18] Eteng MU, Eyong EU, Akpanyung EO, Agiang MA, Aremu CY (1997). Recent advances in caffeine and theobromine toxicities: a review. Plant Foods Hum Nutr.

[19] Zini A, Albert O, Robaire B (2014). Assessing sperm chromatin and DNA damage: clinical importance and development of standards. Andrology.

[20] Zini A, Bach PV, Al-Malki AH, Schlegel PN (2017). Use of testicular sperm for ICSI in oligozoospermic couples: how far should we go?. Hum Reprod.

[21] Bach PV, Schlegel PN (2016). Sperm DNA damage and its role in IVF and ICSI. Basic Clin Androl.

[22] Marshburn PB, Sloan CS, Hammond MG (1989). Semen quality and association with coffee drinking, cigarette smoking, and ethanol consumption. Fert Ster.

[23] Oldereid NB, Rui H, Purvis K (1992). Lifestyles of men in barren couples and their relationships to sperm quality. Eur J Obstet Gynecol Rep Biol.

[24] Parazzini F, Marchini M, Tozzi L, Mezzopane R, Fedele L (1993). Risk factors for unexplained dyspermia in infertile men: a case-control study. Arch Androl.

[25] Florack EI, Zielhuis GA, Rolland R (1994). Cigarette smoking, alcohol consumption, and caffeine intake and fecundability. Prev Med.

[26] Figà-Talamanca I, Cini C, Varricchio GC, Dondero F, Gandini L, Lenzi A (1996). Effects of prolonged autovehicle driving on male reproduction function: a study among taxi drivers. Am J Ind Med.

[27] Curtis KM, Savitz DA, Arbuckle TE (1997). Effects of cigarette smoking, caffeine consumption, and alcohol intake on fecundability. Am J Epidemiol.

[28] Vine MF, Setzer RW, Everson RB, Wyrobek AJ (1997). Human sperm morphometry and smoking, caffeine, and alcohol consumption. Reprod Toxicol.

[29] Robbins WA, Vine MF, Truong KY, Everson RB (1997). Use of fluorescence in situ hybridization (FISH) to assess effects of smoking, caffeine, and alcohol on aneuploidy load in sperm of healthy men. Environ Mol Mutagen.

[30] Jensen TK, Henriksen TB, Hjollund NH, Scheike T, Kolstad H, Giwercman A (1998). Caffeine intake and fecundability: a follow-up study among 430 Danish couples planning their first pregnancy. Reprod Toxicol.

[31] Klonoff-Cohen H, Bleha J, Lam-Kruglick P (2002). A prospective study of the effects of female and male caffeine consumption on the reproductive endpoints of IVF and gamete intra-Fallopian transfer. Hum Reprod.

[32] Horak S, Polanska J, Widlak P (2003). Bulky DNA adducts in human sperm: relationship with fertility, semen quality, smoking, and environmental factors. Mutat Res.

[33] Sobreiro BP, Lucon AM, Pasqualotto FF, Hallak J, Athayde KS, Arap S (2005). Semen analysis in fertile patients undergoing vasectomy: reference values and variations according to age, length of sexual abstinence, seasonality, smoking habits and caffeine intake. Sao Paulo Med J.

[34] Cole DC, Wainman B, Sanin LH, Weber JP, Muggah H, Ibrahim S (2006). Environmental contaminant levels and fecundability among non-smoking couples. Reprod Toxicol.

[35] Schmid TE, Eskenazi B, Baumgartner A, Marchetti F, Young S, Weldon R (2007). The effects of male age on sperm DNA damage in healthy non-smokers. Hum Reprod.

[36] Kobeissi L, Inhorn MC. Health issues in the Arab American community. Male infertility in Lebanon: a case-controlled study. Ethn Dis 2007;17(2 Suppl 3):S3-33-S3-38. 17985448

[37] Ramlau-Hansen CH, Thulstrup AM, Bonde JP, Olsen J, Bech BH (2008). Semen quality according to prenatal coffee and present caffeine exposure: two decades of follow-up of a pregnancy cohort. Hum Reprod.

[38] Jensen TK, Swan SH, Skakkebaek NE, Rasmussen S, Jørgensen N (2010). Caffeine intake and semen quality in a population of 2,554 young Danish men. Am J Epidemiol.

[39] Wogatzky J, Wirleitner B, Stecher A, Vanderzwalmen P, Neyer A, Spitzer D (2012). The combination matters--distinct impact of lifestyle factors on sperm quality: a study on semen analysis of 1683 patients according to MSOME criteria. Reprod Biol Endocrinol.

[40] Jurewicz J, Radwan M, Sobala W, Radwan P, Jakubowski L, Hawuła W (2014). Lifestyle factors and sperm aneuploidy. Reprod Biol.

[41] Yang H, Chen Q, Zhou N, Sun L, Bao H, Tan L (2015). Lifestyles Associated With Human Semen Quality: Results From MARHCS Cohort Study in Chongqing, China. Medicine (Baltimore).

[42] Radwan M, Jurewicz J, Merecz-Kot D, Sobala W, Radwan P, Bochenek M (2016). Sperm DNA damage-the effect of stress and everyday life factors. Int J Impot Res.

[43] Wesselink AK, Wise LA, Rothman KJ, Hahn KA, Mikkelsen EM, Mahalingaiah S (2016). Caffeine and caffeinated beverage consumption and fecundability in a preconception cohort. Repr Toxicol.

[44] Adelusi B, Al-Twaijiri MH, Al-Meshari A, Kangave D, Al-Nuaim LA, Younnus B (1998). Correlation of smoking and coffee drinking with sperm progressive motility in infertile males. Afr J Med Med Sci.

[45] Al-Inany HG, Dumoulin JCM, Evers JLH (2001). Does bicycling really affect semen quality?. Middle East Fert Soc J.

[46] Belloc S, Cohen-Bacrie M, Dalleac A, Amar E, Hazout A, de Mouzon J. Caffeine intake and sperm parameters. Analysis of a cohort of 4474 consecutive semen samples. Fertil Steril. 2013;100(3S1):S212.

[47] Karmon AE, Toth TL, Afeiche MC, Tanrikut C, Hauser R, Chavarro JC. Alcohol and caffeine intake in relation to semen parameters among fertility patients. Fertil Steril. 2013;100(3S1):S12.

[48] Karmon AE, Toth TL, Gaskins AJ, Afeiche MC, Tanrikut C, Hauser R (2014). Male caffeine and alcohol intake in relation to in vitro fertilization outcome among fertility patients. Fertil Steril.

[49] Pecoraro A, Boeri L, Galdini A, Scano R, Ventimiglia E, Serino A (2015). Dietary habits and reproductive health – results of a sociological case-control study. Hum Rep.

[50] Sotomayor RE, Sega GA (2000). Unscheduled DNA synthesis assay in mammalian spermatogenic cells: an update. Environ Mol Mutagen.

[51] Beshay VE, Bukulmez O (2012). Sperm DNA damage: how relevant is it clinically?. Curr Opin Obstet Gynecol.

[52] Brown J, Kreiger N, Darlington GA, Sloan M (2001). Misclassification of exposure: coffee as a surrogate for caffeine intake. Am J Epidemiol.

[53] Gabrielsen JS, Tanrikut C (2016). Chronic exposures and male fertility: the impacts of environment, diet, and drug use on spermatogenesis. Andrology.

[54] Giahi L, Mohammadmoradi S, Javidan A, Sadeghi MR (2016). Nutritional modifications in male infertility: a systematic review covering 2 decades. Nutr Rev.

[55] Nargund VH. Effects of psychological stress on male fertility. Nat Rev Urol. 2015;12:373–82. 10.1038/nrurol.2015.112 26057063

[56] Malik VS, Popkin BM, Bray GA, Després JP, Willett WC, Hu FB (2010). Sugar-sweetened beverages and risk of metabolic syndrome and type 2 diabetes: a meta-analysis. Diabetes Care.

[57] Malik VS, Schulze MB, Hu FB (2006). Intake of sugar-sweetened beverages and weight gain: a systematic review. Am J Clin Nutr.

[58] Wise LA, Rothman KJ, Mikkelsen EM, Sørensen HT, Riis A, Hatch EE (2010). An internet-based prospective study of body size and time-to-pregnancy. Hum Reprod.

[59] Chiu YH, Afeiche MC, Gaskins AJ, Williams PL, Mendiola J, Jørgensen N (2014). Sugar-sweetened beverage intake in relation to semen quality and reproductive hormone levels in young men. Hum Reprod.

[60] Hassold TJ, Abruzzo M, Adkins K, Griffin D, Merrill M, Millie E (1996). Human aneuploidy: incidence, origin, and etiology. Environ Mol Mutagen.

[61] Jurewicz J, Hanke W, Radwan M, Bonde JP (2009). Environmental factors and semen quality. Int J Occup Med Environ Health.

[62] Sánchez-Pozo MC, Mendiola J, Serrano M, Mozas J, Björndahl L, Menkveld R (2013). et a. Special Interest Group in Andrology of the European Society of Human Reproduction and Embriology. Proposal of guidelines for the appraisal of SEMen QUAlity studies (SEMQUA). Hum Reprod.

[63] Björndahl L, Barratt CL, Mortimer D, Jouannet P (2016). 'How to count sperm properly': checklist for acceptability of studies based on human semen analysis. Hum Reprod.

